# A Rare Masquerade: Primary Parotid Lymphoma Mimicking a Parotid Neoplasm in a Young Female Patient

**DOI:** 10.7759/cureus.110760

**Published:** 2026-06-12

**Authors:** Asif Ali, Azif Ali Usman, Joicy Els Jojo, Jose Gamalial

**Affiliations:** 1 Department of General Surgery, MES Academy of Medical Sciences, Perinthalmanna, IND

**Keywords:** lymphoma, parotid glands, parotid neoplasm, surgical case report, total conservative parotidectomy

## Abstract

Primary parotid lymphoma can closely mimic epithelial salivary gland tumors, making preoperative diagnosis challenging. We report the case of a 21-year-old female patient who presented with a left parotid swelling. Imaging was suggestive of a parotid neoplasm, and ultrasound-guided fine needle aspiration cytology (FNAC) demonstrated atypia of undetermined significance (Milan category III). The patient underwent a left total conservative parotidectomy. Histopathological examination with immunohistochemistry (IHC) established the diagnosis of diffuse large B-cell lymphoma (DLBCL), non-germinal center subtype, with involvement of the medial resection margin. She subsequently underwent staging evaluation and received systemic chemotherapy, with a favorable clinical response, and is currently disease-free on follow-up. This case highlights the diagnostic challenges associated with primary parotid lymphoma and underscores the importance of continued reporting of such cases to support the development of improved preoperative diagnostic strategies and management protocols.

## Introduction

Primary non-Hodgkin lymphoma (NHL) of the salivary glands is uncommon, accounting for nearly 2% of all salivary gland neoplasms [1]. Among the salivary glands, the parotid gland is most frequently involved (79%), followed by the submandibular gland (18%), minor salivary glands (2%), and the sublingual gland (1%) [2]. Because primary parotid lymphoma often presents as a painless, slowly enlarging mass with non-specific clinical and radiological features, it may closely mimic benign and malignant parotid epithelial tumors and is therefore rarely suspected preoperatively. Unlike most parotid tumors, which are typically managed surgically through procedures such as superficial or total parotidectomy, primary parotid lymphomas are primarily treated with chemotherapy and/or radiotherapy. Unfortunately, parotid lymphoma is often diagnosed postoperatively in histopathological analysis.

We report a case of extranodal diffuse large B-cell lymphoma (DLBCL) of the parotid gland in a 21-year-old female patient who presented with a progressively growing parotid mass. It was initially suspected to be a benign tumor but was later diagnosed as lymphoma after total conservative parotidectomy. Given that primary parotid DLBCL predominantly affects middle-aged and elderly individuals [3], the occurrence of this tumor in a young patient makes this case particularly unusual and clinically instructive.

## Case presentation

A 21-year-old female patient presented with a painless swelling in front of the left earlobe for one month. The swelling was initially small and rapidly increased in size two weeks before presentation. There was no history of pain, fever, or constitutional symptoms. There was no history or symptoms suggestive of sialolithiasis or malignancy.

On examination, a firm non-tender swelling measuring 4 × 4 cm was noted extending vertically from the tragus of the left ear to just above the angle of the mandible (Figure 1). The surface appeared nodular. The swelling was mobile vertically but with restricted horizontal mobility. The deep lobe was not palpable on bidigital palpation. Stensen’s duct opening appeared normal with no discharge. Facial nerve examination was normal. No other neck swellings were noted. Other salivary glands appeared normal. Laboratory tests showed no abnormalities.

**Figure 1:**
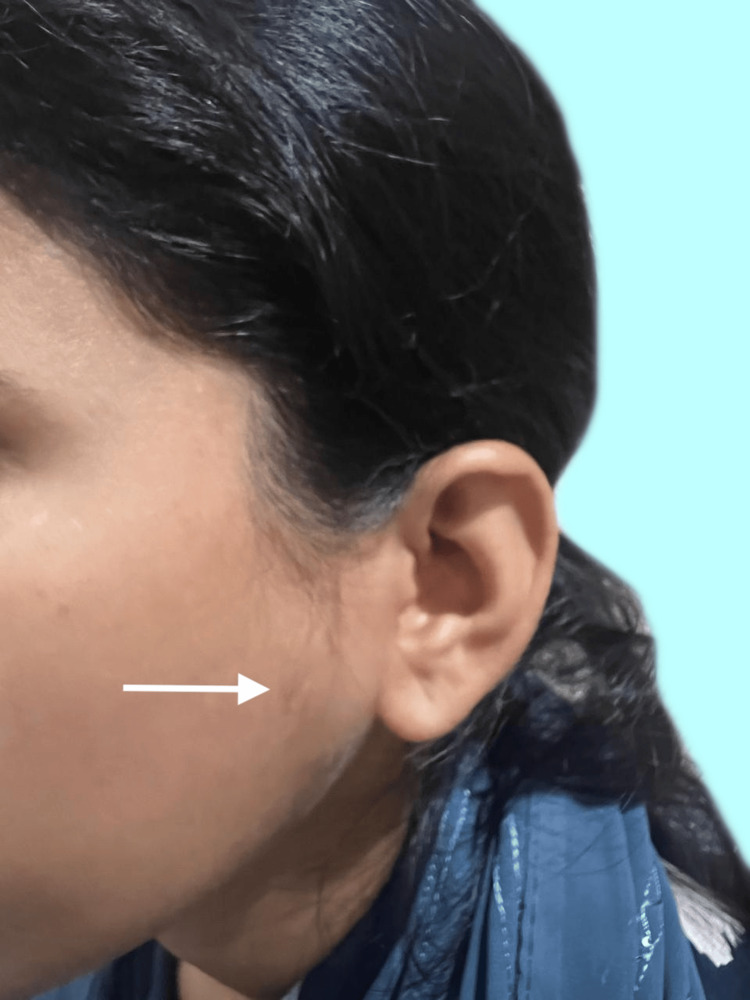
The 21-year-old female patient with a 4x4 cm non-tender swelling in the left parotid area (white arrow).

Ultrasound of the neck showed an ill-defined lobulated hypoechoic lesion in the superficial lobe of the left parotid gland measuring 27 × 16 mm with mild internal vascularity on color doppler, which was suggestive of left parotid gland neoplasm. Ultrasound-guided fine needle aspiration cytology (FNAC) showed atypia of undetermined significance corresponding to Milan category III.

MRI of the neck with gadolinium contrast showed a well-defined, smoothly marginated, partially exophytic lesion measuring approximately 2.7 × 2.6 × 3.7 cm involving the superficial lobe of the left parotid gland. The lesion appeared hypointense on T1, iso to hyperintense on T2, and hyperintense on short tau inversion recovery (STIR) sequences with mild heterogeneous post-contrast enhancement (Figure 2). No deep lobe involvement was noted. No significant cervical lymphadenopathy was identified on MRI. These findings were also suggestive of a left parotid gland neoplasm.

**Figure 2:**
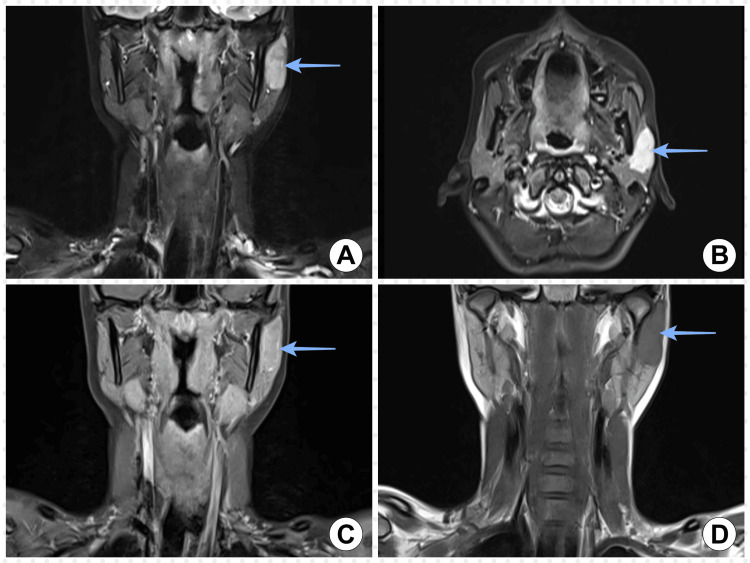
Preoperative MRI showing a well-defined, smoothly marginated, partially exophytic focal lesion measuring approximately 2.7 x 2.6 x 3.7 cm involving the anterior two-thirds of the superior half of the superficial lobe of the left parotid gland (blue arrows). (A) Coronal section showing T2/STIR hyperintense signals. (B) Axial section showing T2/STIR hyperintense signals. (C) Coronal section demonstrating mild post-contrast enhancement. (D) Coronal section demonstrating a well-defined T1 hypointense lesion involving the superficial lobe. STIR: short tau inversion recovery

The patient was planned for a left superficial parotidectomy, but intraoperatively, it was decided to proceed with a left total conservative parotidectomy. Intraoperatively, a hard tumor with dense adhesions was noted in the superior aspect of the superficial lobe of the left parotid gland, which was extending into the deep lobe. The deep lobe of the gland was excised in pieces and sent separately, along with the main specimen, for histopathological analysis. The facial nerve trunk and its branches were identified and preserved (Figure 3). The retromandibular vein was ligated and divided.

**Figure 3:**
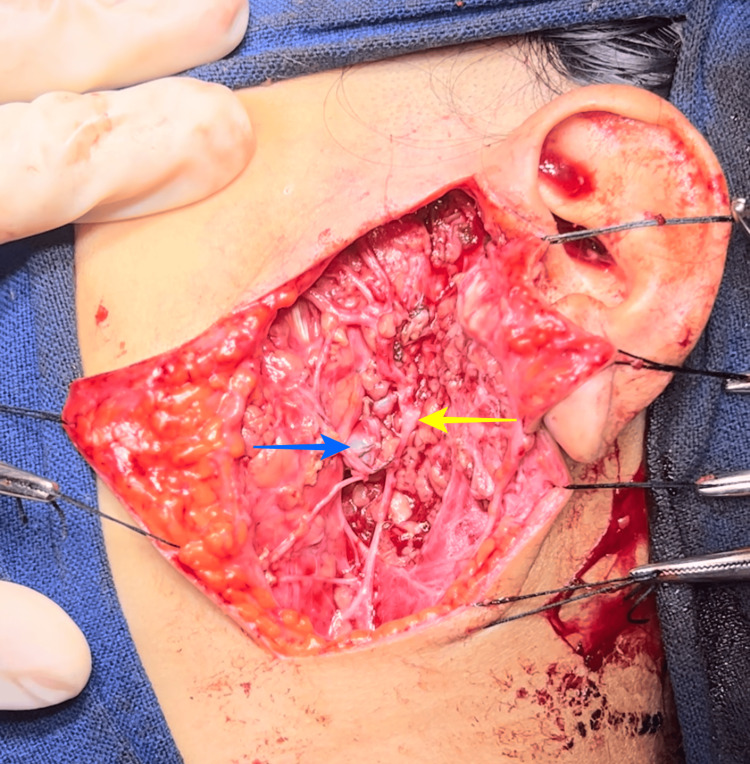
Intraoperative photograph after total conservative parotidectomy showing preserved facial nerve and its branches (yellow arrow) and ligated retromandibular vein (blue arrow).

Microscopically, a fairly circumscribed neoplasm with complete effacement and loss of architecture was identified, composed of large atypical lymphoid cells with moderate eosinophilic cytoplasm and vesicular chromatin, showing one to two nucleoli and moderate nuclear pleomorphism (Figure 4). The medial resected margin was involved in the neoplasm. On additional immunohistochemical (IHC) staining, malignant lymphoid cells were positive for CD20, Bcl-6, and MUM1, showing a high Ki67 proliferative index of 98% (Figure 4). Based on these IHC features, the overall results were consistent with DLBCL, non-germinal center subtype.

**Figure 4:**
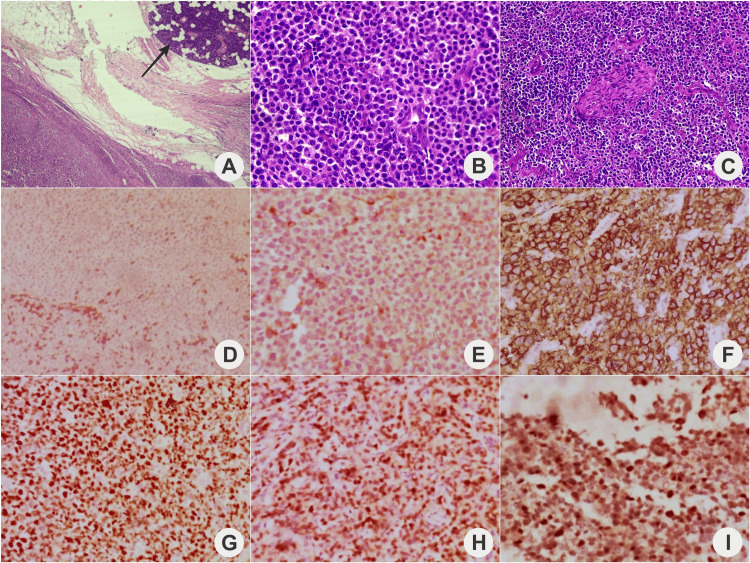
Histopathological stainings and immunohistochemical examinations. A: Overview (hematoxylin and eosin, 4x) showing tumor adjacent to normal parotid gland tissue. B: Large atypical lymphoid cells with moderate eosinophilic cytoplasm and vesicular chromatin showing prominent nucleoli with moderate nuclear pleomorphism (hematoxylin and eosin, 40x). C: Perineural invasion by tumor cells (hematoxylin and eosin, 20x). D: Immunohistochemical negativity for CD3 (10x). E: Immunohistochemical negativity for CD10 (40x). F: Immunohistochemical strong diffuse membranous positivity for CD20 (40x). G: Immunohistochemical positivity for Bcl6 (40x). H: Immunohistochemical positivity for MUM1 (40x). I: Ki67 shows a high proliferation rate of 98% (40×).

The postoperative period was uneventful. Following histopathological diagnosis, the patient was referred to a tertiary oncology center for staging and further management. After an appropriate staging evaluation, she received six cycles of rituximab, cyclophosphamide, doxorubicin, vincristine, and prednisone (R-CHOP) chemotherapy. At the latest follow-up, four months after surgery, she remained asymptomatic with no clinical evidence of disease recurrence.

## Discussion

Primary NHL of the parotid gland is uncommon, accounting for approximately 1% to 4% of all parotid tumors. The most frequently reported histological subtypes include extranodal marginal zone B-cell lymphoma of mucosa-associated lymphoid tissue (MALT), follicular lymphoma, and DLBCL, all of which arise from B lymphocytes [4]. Clinically, patients most often present with a slowly progressive, painless, solitary swelling in the parotid region [4]. Since the clinical features are non-specific, it is rarely considered in the preoperative differential diagnoses.

Autoimmune disorders involving the salivary glands are known to predispose patients to the development of NHL, likely due to chronic antigenic stimulation leading to uncontrolled lymphoid proliferation [5]. In such cases, NHL often represents a monoclonal proliferation of B cells arising from pre-existing polyclonal lymphoid infiltrates, as seen in benign lymphoepithelial lesions, which are also found in Sjögren’s syndrome [6]. Sjögren’s syndrome, in particular, has been strongly linked to an increased risk of salivary gland lymphoma, with studies reporting a 6.5-fold increased risk of NHL overall, a 250-fold increased risk of parotid gland NHL, and nearly a 1,000-fold increased risk of parotid gland MALT lymphoma [7]. However, in the present case, the patient did not exhibit any clinical features suggestive of an underlying autoimmune disorder.

FNAC plays an important role in the initial evaluation of salivary gland tumors; however, its diagnostic utility is limited in cases of malignant lymphoma. Fakhry et al. reported a sensitivity of 80% and specificity of 89.5% for FNAC in diagnosing salivary gland malignancies [8]. False negative results are not uncommon, particularly in lesions such as Warthin's tumor, pleomorphic adenoma, and lymphoepithelial lesions [4]. In lymphoma, the diagnostic yield of FNAC may be further reduced due to inadequate cellularity and the heterogeneous nature of lymphoid proliferations, leading to inconclusive or false-negative findings. This limitation was also observed in our case, where FNAC revealed atypia of undetermined significance. Additionally, FNAC specimens are often insufficient for detailed immunohistochemical analysis, which is essential for definitive diagnosis and subtyping of lymphoma.

Imaging findings in parotid lymphoma are often non-specific and may mimic both benign lesions, such as pleomorphic adenoma, and other malignant neoplasms [4]. Computed tomography is commonly used as the initial imaging modality because it is cost-effective, widely available, and provides rapid and valuable information regarding tumor size, location, and involvement of adjacent structures. MRI can further aid in the evaluation of parotid neoplasms.

Previous studies have reported that head and neck lymphomas typically demonstrate low signal intensity on T1-weighted images and variable low to high signal intensity on T2-weighted images, with heterogeneous enhancement following gadolinium administration [9]. These MRI findings were similar to our case. However, these findings are not consistent, and no definitive MRI characteristics have been established for detecting malignant lymphoma of the head and neck region.

A definitive diagnosis of malignant lymphoma relies on histopathological examination supplemented by IHC, which is essential for accurate diagnosis and subclassification. The increasing availability of less invasive diagnostic modalities, such as image-guided core needle biopsy, along with ancillary techniques including flow cytometry and IHC, may improve the likelihood of establishing a preoperative diagnosis [10]. In the present case, a preoperative core needle biopsy was not performed despite the inconclusive FNAC findings. Although core needle biopsy is available at our center, it is not routinely employed for parotid lesions in our institutional practice because it is an invasive procedure that may cause patient discomfort and presents technical challenges due to the proximity of vital neurovascular structures. Furthermore, the lesion was clinically suggestive of a benign superficial lobe tumor, with no features raising suspicion for malignancy. Therefore, surgical excision was considered both diagnostic and therapeutic. The diagnosis of DLBCL was established only on postoperative histopathological examination. We acknowledge that the absence of a preoperative tissue diagnosis with core needle biopsy represents a limitation of the present case.

Once diagnosed, the primary treatment for parotid lymphoma consists of systemic chemotherapy, with radiotherapy considered in selected cases. Surgical intervention is traditionally reserved for diagnostic purposes, although its role in selected patients remains an area of ongoing investigation [11].

DLBCL exhibits biological heterogeneity, and the non-germinal center B-cell (non-GCB) subtype has been associated with less favorable outcomes compared with the germinal center B-cell subtype [12]. Prognosis in DLBCL is influenced by several established factors, including serum lactate dehydrogenase levels, Eastern Cooperative Oncology Group performance status, Ann Arbor stage, and the International Prognostic Index score. In the present case, detailed records regarding these prognostic parameters were unavailable because the patient underwent staging and subsequent oncological management at a tertiary referral center. The absence of these data represents a limitation of the present report. Nevertheless, primary parotid lymphomas are uncommon and, when diagnosed early and treated appropriately, may be associated with favorable outcomes [13].

## Conclusions

Primary parotid DLBCL is an uncommon entity that may closely mimic benign and malignant parotid neoplasms because of its non-specific clinical and radiological features. This case highlights the diagnostic challenges associated with primary parotid lymphoma, particularly in young patients with apparently benign parotid masses and inconclusive cytology. Clinicians should maintain a high index of suspicion and include lymphoma in the differential diagnosis of parotid swellings. Although the diagnosis is often established after surgical excision performed for a presumed parotid neoplasm, timely staging and initiation of systemic therapy remain essential for achieving favorable outcomes. Further studies are needed to optimize diagnostic strategies and establish standardized management protocols for primary parotid lymphoma.
